# Development and Validation of an UHPLC-QqQ-MS Technique for Simultaneous Determination of Ten Bioactive Components in Fangji Huangqi Tang

**DOI:** 10.1155/2016/1435106

**Published:** 2016-05-26

**Authors:** Xiaoli Wang, Xiao Liu, Tingting Zhu, Baochang Cai

**Affiliations:** ^1^School of Pharmacy, Nanjing University of Chinese Medicine, Nanjing 210023, China; ^2^Engineering Center of State Ministry of Education for Standardization of Chinese Medicine Processing, Nanjing University of Chinese Medicine, Nanjing 210023, China; ^3^Nanjing Haichang Chinese Medicine Group Corporation, Nanjing 210061, China

## Abstract

The aim of this study is to develop an ultrahigh performance liquid chromatography method coupled with triple quadrupole mass spectrometry for simultaneous determination of tetrandrine, fangchinoline, atractylenolide I, atractylenolide III, calycosin-7-O-*β*-D-glucoside, glycyrrhizin, liquiritin, isoliquiritin, liquiritigenin, and isoliquiritigenin in Fangji Huangqi Tang (FHT). The chromatographic separation was performed on a reversed-C_18_ column, eluted with a mixture of 0.1% acetic acid and acetonitrile at 0.4 mL/min. The separation of these ten compounds was achieved by linear gradient elution. The method was strictly validated with respect to specificity, precision, accuracy, and repeatability. All the compounds showed good linearities (*r* ≥ 0.999). The LOQs of the ten components were 0.36, 0.18, 0.09, 0.43, 0.02, 1.89, 0.26, 0.18, 0.61, and 0.48 ng/mL for tetrandrine, fangchinoline, atractylenolide I, atractylenolide III, calycosin-7-O-*β*-D-glucoside, glycyrrhizin, liquiritin, isoliquiritin, liquiritigenin, and isoliquiritigenin, respectively. The LODs of the ten components were 0.11, 0.05, 0.03, 0.13, 0.01, 0.57, 0.08, 0.05, 0.18, and 0.14 ng/mL for tetrandrine, fangchinoline, atractylenolide I, atractylenolide III, calycosin-7-O-*β*-D-glucoside, glycyrrhizin, liquiritin, isoliquiritin, liquiritigenin, and isoliquiritigenin, respectively. The method was proven to be specific and reliable, which would provide a meaningful basis for the quality control and evaluation of FHT during its clinical application.

## 1. Introduction

Fangji Huangqi Tang (FHT) is a classical formula of traditional Chinese medicine (TCM). It was firstly recorded in “Jing Gui Yao Lue” by Zhongjing Zhang in the Han Dynasty. The formula consisted of four herbs, including* Stephania tetrandra* S. Moore,* Astragalus membranaceus* (Fisch.) Bge.,* Atractylodes macrocephala* Koidz., and* Glycyrrhiza uralensis* Fisch. Lots of doctors recommended FHT for the treatment of chronic glomerulonephritis, cardiac edema, and rheumatic arthritis in Chinese clinical application based on their actual experience [1]. Also, experts began to do their research work to reveal the potential effective compounds in FHT [2]. For safe and effective use of FHT in the clinic, quality control and evaluation of this formula with multiple compounds' identification and determination became essential and important. However, to our knowledge, there are no relative reports until now. It has already been found out that FHT contains different kinds of components, such as flavonoids (liquiritigenin, isoliquiritigenin, liquiritin, formononetin, and calycosin), alkaloids (fangchinoline and tetrandrine), lactones (atractylenolide I, atractylenolide II, atractylenolide III, and atractylon), and saponins (astragaloside I, astragaloside II, astragaloside III, and astragaloside IV) [3]. Among these compositions, fangchinoline, tetrandrine, and liquiritigenin, as well as calycosin, all showed good effects on anti-inflammatory and improving immunity [4–7]. The critical strategy for quality control and evaluation of FHT should be applied to study as much of these effective components as possible.

Several common analytical techniques were usually used for multiple chemical compounds' determination in TCM, including HPLC-DAD, UV, HPLC-ELSD, and LC-ICPMS [8–14]. However, these methods showed lots of drawbacks and technical shortcomings, such as time-consuming sample running, poor sensitivity, peak interferences, and limitation of detectors on components identification. As it is widely accepted, the UHPLC-QqQ-MS technique has a special advantage of high resolution and acute sensitivity. And it is now a very powerful tool for the determination of complex chemical compounds in TCM prescriptions, by which the identification of each analyte is also accomplished at the same time. For this reason, a simple, rapid, and sensitive method for simultaneous determination of 10 components in FHT was developed based on UHPLC-QqQ-MS technique in this paper, and the feasibility of this technique on TCM quality control and evaluation was strongly demonstrated.

## 2. Experimental

### 2.1. Reagents and Materials

*Astragalus membranaceus* (Fisch.) Bge. and* Glycyrrhiza uralensis* Fisch. were obtained from Nanjing Haichang Chinese Medicine Group Corporation (Nanjing, China).* Stephania tetrandra* S. Moore and* Atractylodes macrocephala* Koidz. were obtained from Hebei Chinese Medicine Group Corporation (Hebei, China). Fangchinoline, tetrandrine, calycosin-7-O-*β*-D-glucoside, atractylenolide I, atractylenolide III, liquiritin, isoliquiritin, liquiritigenin, isoliquiritigenin, and glycyrrhizin with purity of 99% or higher were purchased from the Sichuan Victor Biological Technology Co. (Chengdu, China). HPLC-grade methanol and acetonitrile were purchased from E. Merck (Merck, Darmstadt, Germany). Purified water was from a Milli-Q system (Millipore Corporation, Bedford, MA, USA). Formic acid was of analytical grade and was obtained from Nanjing Chemical Reagent Company (Nanjing, China).

### 2.2. Instruments

The UHPLC system (Shimadzu, Kyoto, Japan) consisted of LC-30AD binary pump, an autosampler (Model SIL-30SD), an online degasser (DGU-20A5R), and a column temperature controller compartment (CTO-30A). An Extend-C_18_ column (Agilent, USA, 2.1 mm × 100 mm, 1.8 *μ*m) at a temperature of 30°C was used for separation. Mass spectrometric detection was performed using triple quadrupole 5500 instrument (AB Sciex, Concord, Ontario, Canada) equipped with an electrospray ionization (ESI) source.

### 2.3. UHPLC-MS/MS Conditions

The separation of these ten compounds was achieved by linear gradient elution using a mobile phase consisting of 0.1% (v/v) aqueous formic acid (A) and acetonitrile (B). The gradient program was as follows: 0.1–0.8 min, 5% B–50% B; 0.8–3 min, 50% B–90% B; 3–3.1 min, 90% B–5% B. The flow rate was set at 0.4 mL/min. The samples were placed in an autosampler at 4°C prior to injection and the injection volume was 10 *μ*L. Both positive and negative ionization mass spectrometric analyses were conducted due to the physicochemical properties of the ten analytes. The scan mode was set as multiple reactions monitoring (MRM) and the selected monitor ions were* m/z* 623.2/174.1 for tetrandrine,* m/z* 609.3/367.1 for fangchinoline,* m/z* 233.1/187.2 for atractylenolide I,* m/z* 249.1/231.0 for atractylenolide III,* m/z* 447.2/285.1 for calycosin-7-O-*β*-D-glucoside,* m/z* 821.3/351.2 for glycyrrhizin,* m/z* 417.1/255.1 for liquiritin,* m/z* 417.1/255.1 for isoliquiritin,* m/z* 256.2/135.0 for liquiritigenin, and* m/z* 256.2/135.0 for isoliquiritigenin. Also, another selected monitor ion pair was selected for the identification purpose for each analyte. They were* m/z* 623.2/181.3 for tetrandrine,* m/z* 609.3/192.2 for fangchinoline,* m/z* 233.1/215.1 for atractylenolide I,* m/z* 249.1/163.0 for atractylenolide III,* m/z* 447.2/270.0 for calycosin-7-O-*β*-D-glucoside,* m/z* 821.3/193.1 for glycyrrhizin,* m/z* 417.1/135.0 for liquiritin,* m/z* 417.1/135.0 for isoliquiritin,* m/z* 256.2/119.1 for liquiritigenin, and* m/z* 256.2/119.1 for isoliquiritigenin. Source condition parameters were as follows: curtain gas set at 35, ion source temperature adjusted to 550°C, and ion source gas 1 (GS 1) and ion source gas 2 (GS 2) both at 55.

### 2.4. Preparation of FHT Sample

According to the original composition and preparation process of FHT recorded in “Jin-Gui-Yao-Lue,” FHT was obtained in the following procedure: pieces of* Stephania tetrandra* S. Moore (12 g),* Astragalus membranaceus* (Fisch.) Bge. (15 g),* Atractylodes macrocephala* Koidz. (9 g), and* Glycyrrhiza uralensis* Fisch. (6 g) were mixed together in a ratio (4 : 5 : 3 : 2, w/w/w/w) and macerated in deionized water for 30 min before being decocted twice with boiling water (1 : 10, w/v and 1 : 8, w/v, resp.), 20 min for each time. The solution was then filtered through a four-layer mesh and was diluted with water to 1000 mL. 2 mL of the above solution was added to 50 mL vessel, and 15 mL of 60% methanol solution was added for ultrasonic extraction. Then, moderate volume of 60% methanol solution was added to the scale of the 50 mL vessel. The solution was centrifuged at 12,000 rpm for 5 min and was filtered through 0.22 *μ*m membrane. The filtrate collected was then used for UHPLC-QqQ-MS analysis.

### 2.5. Calibration Curves and Limits of Detection and Quantification

**Figure 1:**
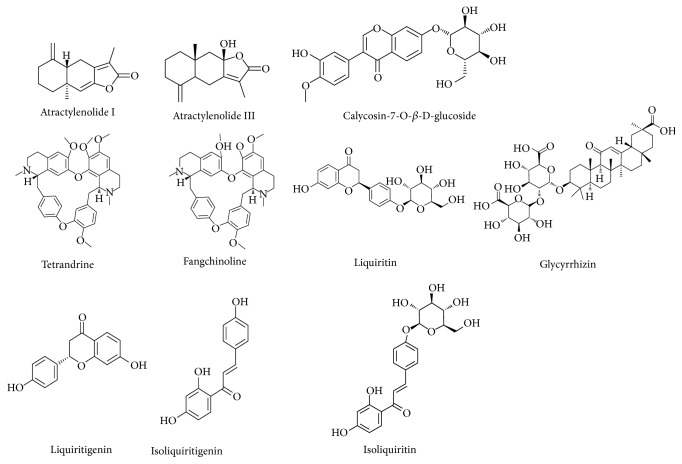
The chemical structures of the ten selected major compounds of FHT.

Stock solutions of the 10 analytes were separately prepared by diluting the reference standards with methanol. The chemical structures of the compounds were shown in Figure 1. All the stock solutions were separately kept at 4°C. A mixed working solution was prepared before use every time. All the standards were soluble in methanol and completely dissolved in the mixed working solution. And the mixed working solution containing 10 compounds was diluted to appropriate concentration ranges for the construction of calibration curves. The calibration curve of each compound was performed with at least seven appropriate concentrations levels. The concentrations of the 10 components in Fangji Huangqi Tang should be included in the range of the standard curve. The limit of detection (LOD) and limit of quantification (LOQ) under the present chromatographic conditions were determined at a signal-to-noise (S/N) ratio of 3 and 10, respectively.

### 2.6. Precision, Accuracy, and Stability

Intraday variations within one day were all taken into consideration to determine the precision of the developed method. The relative standard deviation (RSD) was taken as a measurement of repeatability. A recovery test was utilized to evaluate the accuracy of this method. Certain amounts of 10 standards were accurately added to a FHT sample and then processed and analyzed as described in Sections 2.2 and 2.3. The total amount of each analyte was calculated from the corresponding calibration curve, and the recovery of each analyte was calculated by the following equation: recovery (%) = (amount_determined_ − amount_original_)/amount_spiked_ × 100%, where amount_determined_ is the determined total amount of each analyte, amount_original_ is the original amount of each analyte in samples determined, and amount_spiked_ is the spiked amount of each analyte with 80%, 100%, and 120% ratio of the original amount. In order to investigate the stability of the processed sample solution, the same sample solution was stored at 4°C and analyzed every 12 h for 1 day. In addition, the 10 stock solutions were all determined every week to investigate whether obvious degradation had happened under their preservation condition.

## 3. Results and Discussion

### 3.1. Optimization of Sample Preparation and Experimental Conditions

According to the published papers, many different kinds of components were identified in FHT including alkaloids, flavonoid, and lactones. Also, these chemical compounds were proved to be the main effective components in FHT. The ten selected compounds for simultaneous determination belonged to different chemical classes and covered a wide polarity range. So, it is of great significance to screen out an appropriate sample preparation method based on the effective extraction for various effective compounds. Therefore, a novel sample method has been developed to collect different polar components on a solid phase extraction column. This simple and convenient technique was suitable for traditional Chinese medicine prescriptions detection by enhancing the sample polarity scope, as well as reducing the pollution of the instrument.

It was difficult for researchers to perform good separation for the chemical constituents in FHT because of the complexity of the composition and the existence of isomers. In this paper, the UHPLC conditions were well optimized, including the type of column, column temperature, mobile phase system, and flow rate. After a comparison of different brands of columns including Agilent C_18_, Waters C_18_, and Thermo C_18_, Agilent column finally gave out sound separation for the ten compounds to be determined. In addition, different kinds of mobile phase systems such as acetonitrile and methanol with a variety of modifiers (including formic acid and acetic acid) were tested. As a result, a solution of 0.1% (v/v) aqueous formic acid and acetonitrile at 0.4 mL/min was used as the optimal chromatographic elution system.

Typical source conditions were used as follows: curtain gas was set at 35; ion source temperature was adjusted to 500°C; ion source gas 1 (GS 1) and ion source gas 2 (GS 2) were both set at 55. Independent parameters for the detection of each compound were optimized by injecting standard solution into MS system under MRM mode, including the declustering potential (DP), the collision energy (CE), the entrance potential (EP), and the collision cell exit potential (CXP). These parameters for the detection of the ten constituents were shown in Table 1.

### 3.2. Method Validation

**Figure 2:**
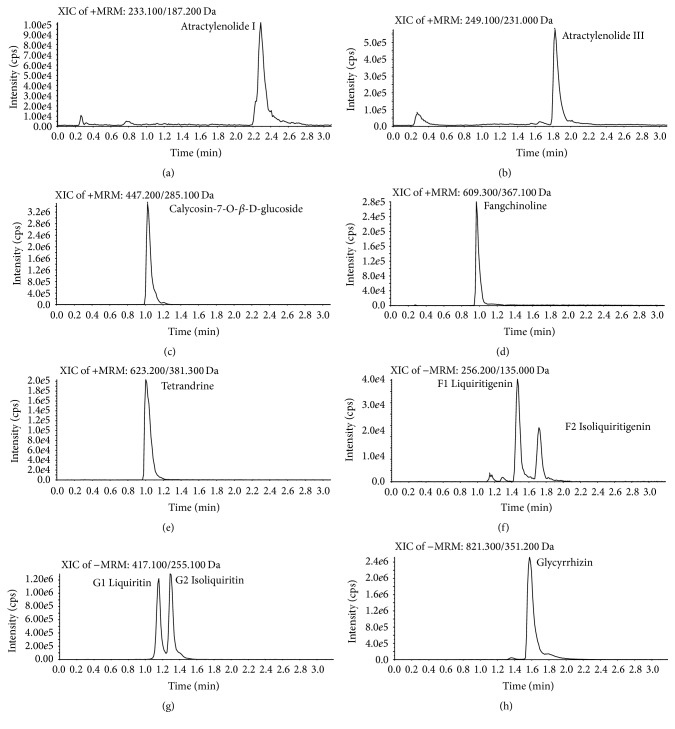
Representative extract ions chromatograms for the determination of the 10 analytes. (a) Atractylenolide I (2.28 min). (b) Atractylenolide III (1.83 min). (c) Calycosin-7-O-*β*-D-glucoside (1.04 min). (d) Fangchinoline (0.98 min). (e) Tetrandrine (1.01 min). (f) Liquiritigenin (1.47 min) and isoliquiritigenin (1.72 min). (g) Liquiritin (1.17 min) and isoliquiritin (1.31 min). (h) Glycyrrhizin (1.60 min).

Chromatographic peaks of FHT were identified by comparing the retention time with that of each reference compound. The representative extract ions chromatograms of the standard mixture solution were shown in Figure 2. (a) stands for fangchinoline (0.98 min); (b) stands for atractylenolide I (2.28 min); (c) stands for tetrandrine (1.01 min); (d) stands for atractylenolide III (1.83 min); (e) stands for calycosin-7-O-*β*-D-glucoside (1.04 min); (f) stands for liquiritigenin (1.47 min) and isoliquiritigenin (1.72 min); (g) stands for liquiritin (1.17 min) and isoliquiritin (1.31 min); (h) stands for glycyrrhizin (1.60 min). According to the mass-to-electric charge ratio of each compound, all compounds were detected in different channels without interfering with each other.  Table 2 shows the regression equation for each reference compound, as well as the LOD and LOQ values and the mass spectrometry information. All the components showed good linearity (*r* > 0.9991) in a relatively wide concentration range. Tables 3 and 4 provide precision and recovery results of the 10 components, respectively. The intraday variations of the compounds were < 2.53%, the relative standard deviation (RSD) of repeatability test was below 2.68% for all the analytes of interest, and the extraction recoveries of the components ranged from 98.51% to 101.10%. Stability was evaluated by analyzing all the analytes in samples kept at 4°C for 24 h. They were all found to be stable within 24 h (RSD < 3%). As a result, the developed method was proved to be precise, accurate, and sensitive for the simultaneous quantification of the major compounds of FHT.

### 3.3. Sample Analysis

The developed UHPLC-MS/MS method was subsequently applied to analysis and quality evaluation of FHT. The content of these major constituents in FHT was shown in Table 5.

## 4. Conclusions

A rapid UHPLC-MS/MS method was established for the comprehensive analysis of FHT. This method could separate the complex constituents in a short run time. Furthermore, MS/MS proposed high sensitivity which is helpful for the quantification of the low-content compounds. The method was successfully applied to the simultaneous determination of 10 bioactive compounds in FHT. This available, rapid, and reliable method is fit for routine analysis and effective quality control of this Chinese herbal prescription.

## Figures and Tables

**Table 1:** List of selected MRM parameters, including CE, DP, EP, and CXP for the 10 major compounds of Fangji Huangqi Tang.

Compound	CE	DP	EP	CXP
Tetrandrine	53.90	39.86	3.93	10.83
Fangchinoline	49.43	27.13	4.29	27.3
Atractylenolide I	22.61	63.96	6.81	14.00
Atractylenolide III	14.42	54.80	3.14	14.00
Calycosin-7-O-*β*-D-glucoside	16.11	57.00	6.38	14.00
Glycyrrhizin	−52.15	−14.67	−13.72	−22.52
Liquiritin	−23.39	−17.32	−10.15	−21.88
Isoliquiritin	−23.39	−17.32	−10.15	−21.88
Liquiritigenin	−21.65	−65.84	−9.14	−17.00
Isoliquiritigenin	−21.65	−65.84	−9.14	−17.00

**Table 2:** Calibration curves, LOD, and LOQ of the 10 major compounds.

Compounds	Calibration curve	*r*	Linear range (ng/mL)	LOQ (ng/mL)	LOD (ng/mL)	MS/MS (*m*/*z*)	Detected ion
Tetrandrine	*y* = 28104.45*x* + 2073.02	0.9996	1.63–104.16	0.36	0.11	623.2/381.3	[M + H]^+^
Fangchinoline	*y* = 23500.46*x* + 2211.84	0.9991	1.84–117.54	0.18	0.05	609.3/367.1	[M + H]^+^
Atractylenolide I	*y* = 116476*x* + 37509.7	0.9998	0.37–23.55	0.09	0.03	233.1/187.2	[M + H]^+^
Atractylenolide III	*y* = 114683*x* + 86369.7	0.9993	0.56–35.63	0.43	0.13	249.1/231.0	[M + H]^+^
Calycosin-7-O-*β*-D-glucoside	*y* = 835469*x* + 982159	0.9999	9.94–635.92	0.02	0.01	447.2/285.1	[M + H]^+^
Glycyrrhizin	*y* = 8268.92*x* + 177769	0.9991	31.40–2009.56	1.89	0.57	821.3/351.2	[M − H]^−^
Liquiritin	*y* = 9740.06*x* + 3971180	0.9996	123.75–7920.00	0.26	0.08	417.1/255.1	[M − H]^ −^
Isoliquiritin	*y* = 20012.34*x* + 4672350	0.9991	30.38–1944.03	0.18	0.05	417.1/255.1	[M − H]^ −^
Liquiritigenin	*y* = 16315.93*x* + 140.37	0.9995	2.12–135.55	0.61	0.18	256.2/135.0	[M − H]^ −^
Isoliquiritigenin	*y* = 35718.6*x* + 2842.59	0.9998	0.61–39.01	0.48	0.14	256.2/135.0	[M − H]^ −^

**Table 3:** Precision and repeatability for the assay of the 10 major compounds.

Compounds	Concentration (ng/mL)	Precision (*n* = 6)		Repeatability (*n* = 5)	
		Found	RSD (%)	Found	RSD (%)
Tetrandrine	52.08	51.49 ± 0.46	0.89	53.52 ± 1.16	2.15
Fangchinoline	58.77	57.56 ± 0.49	0.84	55.87 ± 0.85	1.52
Atractylenolide I	11.77	11.51 ± 0.26	2.27	12.26 ± 0.26	2.15
Atractylenolide III	17.82	17.47 ± 0.14	0.78	21.60 ± 0.58	2.68
Calycosin-7-O-*β*-D-glucoside	317.96	317.81 ± 0.58	0.18	145.27 ± 1.86	1.28
Glycyrrhizin	1003.78	1003.86 ± 1.18	0.12	1661.02 ± 34.97	2.11
Liquiritin	3960.00	3941.00 ± 46.23	1.17	3303.74 ± 24.75	0.75
Isoliquiritin	972.02	974.07 ± 1.20	0.12	695.73 ± 8.31	1.19
Liquiritigenin	67.78	68.90 ± 0.89	1.29	44.50 ± 0.83	1.86
Isoliquiritigenin	19.51	18.94 ± 0.48	2.53	3.11 ± 0.06	2.03

**Table 4:** Recovery of the 10 major compounds in Fangji Huangqi Tang (*n* = 3).

Compounds	Original amount (ng)	Spiked amount (ng)	Detected amount (ng)	Recovery (%)	RSD (%)
Tetrandrine	1304.50	1045.00	2343.50 ± 2.50	99.24	0.91
		1306.00	2601.00 ± 20.50		
		1567.50	2857.00 ± 18.00		

Fangchinoline	1392.50	1111.50	2480.00 ± 27.50	99.27	1.08
		1389.50	2782.50 ± 5.00		
		1667.00	3057.00 ± 21.50		

Atractylenolide I	294.00	232.00	529.00 ± 2.50	101.1	1.40
		290.00	584.00 ± 5.50		
		348.00	647.50 ± 0.50		

Atractylenolide III	540.00	431.00	965.50 ± 10.00	99.88	1.78
		538.50	1079.00 ± 1.50		
		646.50	1192.00 ± 19.50		

Calycosin-7-O-*β*-D-glucoside	3622.00	2897.00	6512.50 ± 6.00	99.59	0.29
		3621.00	7203.00 ± 14.50		
		4345.50	7973.00 ± 79.00		

Glycyrrhizin	41970.00	33575.00	75216.00 ± 145.00	98.74	0.78
		41969.00	83537.50 ± 362.00		
		50362.50	91399.50 ± 501.00		

Liquiritin	82593.50	66074.00	148436.50 ± 254.50	99.18	0.96
		82592.50	163987.50 ± 1503.50		
		99111.00	181061.00 ± 627.50		

Isoliquiritin	17393.50	13914.50	31167.50 ± 95.50	98.84	0.47
		17393.00	34595.00 ± 80.00		
		20871.50	37982.00 ± 52.00		

Liquiritigenin	1112.50	890.50	1991.00 ± 9.50	98.51	1.18
		1113.00	2209.00 ± 14.50		
		1335.50	2426.00 ± 18.00		

Isoliquiritigenin	79.00	63.00	141.50 ± 1.00	99.55	1.09
		78.50	156.50 ± 0.50		
		94.50	173.50 ± 0.50		

**Table 5:** Content of the 10 major compounds in different batches of Fangji Huangqi Tang (*μ*g/mL).

Number	Tetrandrine	Fangchinoline	Atractylenolide I	Atractylenolide III	Calycosin-7-O-*β*-D-glucoside	Glycyrrhizin	Liquiritin	Isoliquiritin	Liquiritigenin	Isoliquiritigenin
1	1.36	1.36	0.30	0.53	3.57	39.80	82.74	17.36	1.09	0.08
2	1.33	1.40	0.32	0.55	3.54	41.79	83.06	17.57	1.10	0.08
3	1.33	1.37	0.30	0.54	3.65	41.98	82.91	17.16	1.08	0.08
Mean	1.34	1.38	0.31	0.54	3.59	41.19	82.90	17.36	1.09	0.08
SD	0.02	0.02	0.01	0.01	0.06	1.21	0.16	0.21	0.01	0.00

## Abbreviations

UHPLC-QqQ-MS: Ultrahigh performance liquid chromatography method coupled with triple quadrupole mass spectrometry
TCM: Traditional Chinese medicine
HPLC-DAD: High performance liquid chromatography with diode array detection
UV: Ultraviolet
HPLC-ELSD: High performance liquid chromatography with evaporative light scattering detector
LC-ICPMS: Liquid chromatography with inductively coupled plasma mass spectrometry.
